# Intolerance of Uncertainty Mediates the Relationship Between Social Anxiety and Problematic Smartphone Use Severity in College Students

**DOI:** 10.3390/jpm15120599

**Published:** 2025-12-04

**Authors:** Sana Alavinikoo, Elyse F. Hutcheson, Jon D. Elhai

**Affiliations:** 1 Department of Psychology, University of Toledo, 2801 West Bancroft Street, Toledo, OH 43606, USA; 2 Department of Neurosciences and Psychiatry, University of Toledo, 3000 Arlington Avenue, Toledo, OH 43614, USA

**Keywords:** problematic smartphone use, intolerance of uncertainty, social anxiety

## Abstract

**Objectives**: Prior research has found that social anxiety and intolerance of uncertainty (IU) are both related to problematic smartphone use (PSU) severity. However, research about the mediating effect of IU from social anxiety to PSU is limited. **Methods**: We conducted a cross-sectional analysis of self-report online data from 329 college students in the United States, evaluating IU, social anxiety, and PSU through structural equation modeling. **Results**: We found that confirmatory factor analytic models of social anxiety, IU and PSU each fit well. Our overall structural equation model also indicated good fit, and IU acted as a significant mediator of the link between social anxiety and PSU severity. To test model specificity, we compared it with an alternative model that added a direct path from social anxiety to PSU. Although the alternative model showed slightly better fit, the improvement was minimal, and theoretical grounds supported keeping the simpler initial model. **Conclusions**: These results indicate that IU may represent a critical cognitive–affective mechanism linking social anxiety to PSU. PSU might function as a coping mechanism for some individuals to alleviate the negative emotion associated with social anxiety and IU.

## 1. Introduction

Since their invention, smartphones have been widely used around the world, to the point that daily life without them is difficult to imagine. Although smartphones make life more convenient by supporting such activities as online shopping, social communication, education, and entertainment [1], excessive use can result in negative consequences on general health, academic performance, and social connections [2].

Problematic forms of smartphone and Internet use are particularly prevalent among university students and the younger generation, where excessive use has negatively affected various aspects of student life [3,4,5]. For instance, among a large U.S. college student sample (n = 2469), 46.9% met criteria for problematic smartphone use (PSU) [6]. Moreover, PSU has been linked to a higher likelihood of traffic accidents resulting from distraction while driving [7], as well as greater incidence of cyberbullying and phubbing [8]. Beyond these behavioral risks, PSU has also shown strong associations with mental health problems, including general anxiety, depressive symptoms, and social anxiety [9,10,11,12]. Several factors have been suggested as contributing to the emergence of PSU. In the current study, we focus on the role of social anxiety and intolerance of uncertainty in association with PSU.

PSU is conceptualized as a problematic Internet use behavior, characterized by difficulties in regulating smartphone use, which lead to various physical, psychological, and social consequences. Similarly to substance use disorders, PSU involves symptoms such as withdrawal, emotional salience, and tolerance [13,14]. We provide the caveat, however, that PSU is not a disorder defined in DSM-5 or ICD-11, but PSU is nonetheless an important construct that is of concern in society.

An increasing number of studies has shown that social anxiety is strongly associated with PSU [11,15,16]. Social anxiety, or social phobia, refers to the excessive fear that individuals experience in social situations, often driven by apprehension about being observed, scrutinized, or negatively evaluated by others [17]. Because social anxiety is highly aversive, individuals often avoid social interactions to manage discomfort, avoid social missteps, and reduce their anxiety [18,19]. For socially anxious individuals, frequent smartphone use could serve as a habitual means of coping with anxiety, and smartphones and social networking sites may function as alternative means of social engagement [20,21]. Socially anxious individuals may excessively rely on online interaction via smartphones to reduce face-to-face apprehension [22,23]. However, this reliance increases the risk of developing PSU, particularly when individuals are unable to reduce or discontinue their use [24]. Moreover, smartphones may serve as an escape from social interaction, as individuals can turn to their screens during social situations, providing a distraction from the immediate context and a sense of control [19]. Research supports that college students with elevated social anxiety tend to rely on smartphones to cope with negative emotions stemming from interpersonal experiences [25,26]. Taken together, these findings highlight that social anxiety not only increases vulnerability to PSU but may also interact with cognitive factors—such as intolerance of uncertainty—that shape how individuals cope with social distress in the digital age [19].

Intolerance of uncertainty (IU) can be conceptualized as an individual’s tendency to react negatively to ambiguous situations [27]. IU is related to a broad variety of mental disorders like depressive disorders, obsessive–compulsive disorder (OCD), generalized anxiety disorder (GAD), and social anxiety disorder [28,29]. People with social anxiety frequently worry about how others will perceive their self-presentation and whether their behavior will be socially appropriate [30,31]. Moreover, social situations are inherently ambiguous, and outcomes cannot be predicted. Performing well in these contexts requires the ability to tolerate some uncertainty [32]. Research suggests that IU components related to difficulty taking action or avoiding uncertain situations are more strongly related to symptoms of social anxiety [29]. As a result, handling social situations is especially challenging for people with social anxiety, because they often have a strong tendency toward experiencing IU [19,33]. Furthermore, mobile phones can function as a maladaptive coping mechanism to alleviate the distress that stems from uncertainty [34]. Supporting this role, research on pre-service teachers (teachers in training) showed that IU indirectly influenced PSU through heightened rumination and anxiety, highlighting a chain of cognitive and emotional processes that exacerbate PSU risk [35]. In another study of graduate students, IU was associated with PSU severity both directly and indirectly by increasing anxiety and reducing positive coping strategies [36]. Taken together it seems that IU can serve as a mediator between social anxiety and PSU.

While prior studies have reported associations between social anxiety and PSU [15,16], the underlying mechanisms explaining how social anxiety contributes to PSU remain unclear. We were only able to find one study that examined the mediating role of IU between social anxiety and PSU [19]. Using correlational analysis and mediation testing, this study reported that individuals experiencing heightened social anxiety also tended to report greater IU, stronger desire to use phones to alleviate anxiety, and increased smartphone use [19]. However, these findings were limited to correlational and mediation methods, which do not capture the latent cognitive and affective mechanisms underlying these relationships. Moreover, IU has often been viewed mainly as a general feature of anxiety rather than a process that explains how socially anxious people develop problematic smartphone use [37,38].

To our knowledge, no previous study has investigated the mediating role of IU between social anxiety and PSU using latent variable-based structural equation modeling, which allows for a more rigorous investigation of the mediating role of IU and can provide stronger evidence for the mechanisms linking social anxiety to PSU. It should be noted that treating the variables as latent rather than observed leads to greater measurement accuracy and reduced error [39,40]. By addressing this gap, the current study provides a clearer picture of how social anxiety drives PSU through cognitive mechanisms such as IU.

Considering the previous research, our main hypotheses are:

**H1.**  *IU is positively associated with PSU severity*.

**H2.**  *Social anxiety is positively associated with PSU severity*.

**H3.**  *IU mediates the relationship between social anxiety and PSU severity*.

### Theory

The Interaction of Person–Affect–Cognition–Execution (I-PACE) framework is commonly used to conceptualize PSU. According to the I-PACE model [41,42], maladaptive Internet use results from the intersection of individual traits (e.g., emotional reactivity, psychopathology), cognitive processes, affective states, and executive functioning. The I-PACE model also conceptualized these affective and cognitive processes as mechanisms through which predisposing psychopathology contributes to problematic Internet use such as PSU.

Within the framework of the I-PACE model [41,42], social anxiety can be conceptualized as a predisposing variable that heightens sensitivity to ambiguous or evaluative social situations, which are often perceived as threatening [43,44]. Individuals with high social anxiety often experience greater IU, perceiving social interactions as unpredictable and threatening [17,45]. IU can further function as a key cognitive–affective vulnerability linking social anxiety to PSU within the I-PACE framework, where situational triggers (e.g., social uncertainty) interact with predispositional psychopathology (social anxiety) and cognitive–affective factors (IU) leading to smartphone use as a coping mechanism that can ultimately reinforce PSU [19,34,41,46].

This conceptualization supports the view that IU functions as a mediating pathway connecting social anxiety to problematic smartphone engagement [19]. In line with this conceptualization, a study found that IU significantly correlated with PSU tendencies, and PSU may be a coping strategy to reduce negative emotions stemming from uncertainty [34]. Another study, reported that non-social smartphone use served as a mediator between IU and PSU severity, highlighting the role of IU in driving individuals toward using smartphones to cope with uncertainty [46].

## 2. Method

### 2.1. Sample and Procedures

The initial sample included 378 students from a mid-sized public university in the Midwestern United States. The data collection took place from 16 November 2023, to 12 April 2024. Following data screening in R [47], 43 participants were excluded due to careless responding (e.g., over 19 consecutive identical responses), replicated response IDs, and substantial missing data (e.g., >50% of survey responses). We also excluded six participants who did not possess a social media account, an exclusion criterion from the larger project from which we collected these data. The final analytic sample was consisted of 329 participants, aged 18–31 years (M = 19.99, SD = 2.14). The current sample size aligns with those reported in previous PSU research [48].

The sample demographics are summarized as follows: 63.8% reported being born female (n = 210), and 35.3% born male (n = 116). 14.3% identified as LGBTQ+ (n = 47). Regarding racial/ethnic background, 59.5% identified as White or Caucasian (n = 196), 26.4% Asian (n = 87), 14.3% African American (n = 47), and 9.7% Latinx (n = 32). These categories were not independent of each other.

An online cross-sectional design was used in the current study. Participants were recruited from the university’s Sona Systems research pool of introductory psychology students, and received course credit in the form of research participation points. After initial eligibility screening, participants were directed to an online consent form. The one who provided online consent completed the survey (in English) on the PsychData platform, which included demographic items, self-report measures, and debriefing materials.

### 2.2. Measures

Participants provided demographic information, including age, sex assigned at birth, sexual orientation, and race/ethnicity. Additionally, the following measures were used.

#### 2.2.1. Social Phobia Inventory (SPIN)

The SPIN has 17 items and it assesses anxiety related to social performance and observation and was used to evaluate social anxiety symptom severity over the past month [49]. Responses are measured using a 5-point scale from 0 (Not at all) to 4 (Extremely), producing a total score range of 0–68, with higher scores representing more severe symptoms. The SPIN has shown excellent internal consistency and good test–retest reliability as a valid measure of social anxiety severity [49,50]. Coefficient alpha for our sample was 0.93 and Omega was 0.95.

#### 2.2.2. Intolerance of Uncertainty Scale-Short Version (IUS-12)

IUS-12 is a 12-item instrument that assesses the degree of intolerance of uncertainty, using a 5-point Likert scale ranging from 1 (not at all characteristic of me) to 5 (entirely characteristic of me) [51]. The IUS-12 represents a shortened form of the original 27-item Intolerance of Uncertainty Scale [52]. Although the IUS-12 has two subscales/factors (prospective and inhibitory intolerance of uncertainty), researchers commonly use the total summed score [51]; in fact, a study found that a unidimensional factor was more parsimonious than the two-factor solution [53]. The IUS demonstrated good internal reliability (Cronbach’s alpha = 0.91), and its validity has been established through correlations with other measures of depression and anxiety [51]. Coefficient alpha for our sample was 0.91 and Omega was 0.93.

#### 2.2.3. Smartphone Addiction Scale-Short Version (SAS-SV)

The SAS-SV (version 4.3.3) [54] is a 10-item scale measuring PSU, with items rated on a 6-point Likert scale from 1 (strongly disagree) to 6 (strongly agree). For consistency, we phrased items in the first person [55]. A composite score is computed, where higher values correspond to greater PSU. The SAS-SV has shown good reliability and validity [56]. In our sample, Cronbach’s alpha was 0.87 and McDonald’s omega was 0.90.

### 2.3. Statistical Procedure

R software v.4.3.3 [47] was used for cleaning the data and conducting preliminary analysis. We employed several R packages v.4.3.3, including naniar (to examine missing data), pastecs (for descriptive statistics), careless (to identify inattentive responding), mice (for imputing missing data), dplyr (for data cleaning), fmsb (to assess internal consistency), skimr (for frequency tables), sjstats (for ANOVA effect sizes), and apaTables (to generate scale intercorrelations).

Following the mentioned data exclusions, missing item-level responses were estimated and imputed using maximum likelihood (ML) procedures separately for each scale prior to computing total and subscale scores. Item-level imputation was performed when fewer than 50% of responses were missing within a given scale. After computing scale scores, a second round of ML estimation was conducted at the scale level for participants who were missing fewer than 50% of their corresponding scores. Descriptive statistics and bivariate correlations were then examined to evaluate normality assumptions, detect potential outliers, and guide the selection of covariates for subsequent analyses.

Confirmatory factor analyses (CFA) and structural equation modeling (SEM) were conducted with Mplus version 8.11 [57]. Measures used Likert-type response formats with verbal anchors for each option, involving five response options for the IUS-12 and SPIN (six response options for the SAS-SV), reflecting an ordinal rather than continuous response scale. Accordingly, items were treated as ordinal variables, and models were estimated with weighted least squares with mean and variance adjustment (WLSMV). This approach relies on a polychoric correlation matrix and estimates factor loadings via probit regression [58]. Given the ordinal scaling and estimation method, item-level normality indices are not reported, as such statistics are primarily relevant for continuous variables. Model fit was assessed using commonly accepted criteria for excellent fit (with acceptable fit in parentheses): Tucker–Lewis Index (TLI) and Comparative Fit Index (CFI) values > 0.95 (0.90–0.94), root mean square error of approximation (RMSEA) values < 0.06 (0.07–0.08), and standardized root mean square residual (SRMR) values < 0.08 (0.09–0.10) [59]. However, RMSEA should be interpreted with caution, as its accuracy is reduced when applied to ordinal data [60], as conducted in this study.

For scaling purposes, the first unstandardized loading of each scale was fixed to 1. For the SAS-SV, residual error covariances were specified between items 1 and 2 (both reflecting missing school and/or work due to smartphone use) and between items 4 and 5 (both reflecting attachment to one’s smartphone) to account for their similar item content. For the SPIN, residual error covariances were specified between items 1 and 16 (both reflecting anxiety around authority figures), items 5 and 12 (both concerning fear of criticism), items 3 and 8 (both related to parties), and items 4 and 10 (both involving talking to strangers) to account for their overlapping content.

**Figure 1:**
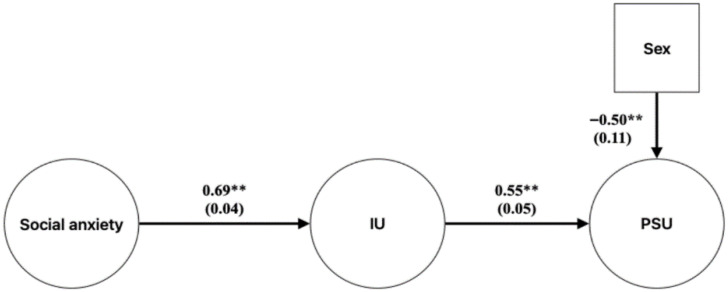
The mediating role of IU between social anxiety and PSU. PSU = problematic smartphone use; IU = intolerance of uncertainty. Path coefficients are presented, with their corresponding standard errors in parentheses. ** indicates *p* < 0.01.

Following the CFAs, we estimated the SEM depicted in Figure 1, employing latent variables to represent our scales. In this model, social anxiety (predictor) was specified to predict IU (mediator), which then in turn was specified to predict PSU (dependent variable). We specified sex as a covariate of PSU [61]. Given the truncated age range of our college sample, we did not model age as a covariate. The mediation was assessed using the delta method for calculating indirect effect standard errors with the WLSMV estimator, using 1000 non-parametric bootstrapped replications.

## 3. Results

### 3.1. Preliminary Analyses

Descriptive statistics and intercorrelations for age and the scale scores are shown in Table 1. All scale scores were significantly correlated with one another. Age was unrelated to scale scores, except for a significant association with PSU severity. Table 2 displays sex differences on the scale scores, showing that sex was significantly associated with all three scale scores, with women scoring higher than men; however, all three effects would be considered small in magnitude.

### 3.2. Individual CFA Results

Table 3 summarizes the CFA results for the three measures, with detailed factor loadings reported in Table A1, Table A2 and Table A3. Consistent with the previously outlined benchmarks, all three CFAs showed adequate to excellent fit, except for the RMSEA index, which, as noted previously, is not an appropriate indicator of model fit for ordinal items [60], such as those used in the present study.

### 3.3. SEM Results

The SEM model, examining the mediating role of the IU factor in the relationship between social anxiety and PSU is presented in Figure 1. The model indicated good fit: robust χ^2^(732) = 1469.51, *p* < 0.001, CFI = 0.94, TLI = 0.94, SRMR = 0.08, RMSEA = 0.05 (90% CI = 0.05–0.06). Figure 1 shows the model’s estimated path coefficients and standard errors. A significant association was found between social anxiety and IU; and IU was significantly related to PSU severity after adjusting for sex. Female sex also was significantly associated with PSU severity.

### 3.4. Mediation Analysis

Mediation analyses are summarized in Table 4, with *p*-values reported to indicate the significance of indirect effects. The path from social anxiety to PSU was significantly mediated by IU.

### 3.5. Model Comparison

To examine whether including an additional direct path between social anxiety to PSU would improve model fit above the model from Figure 1, we tested an alternative model in comparison to the original hypothesized model and compared the two models using a chi-square difference test using Mplus’s DIFFTEST command. The alternative model fit significantly better, χ^2^ diff (1) = 17.84, *p* < 0.001. However, while the original model’s CFI was 0.942, the alternative model’s CFI was only 0.005 points higher (CFI = 0.949). Given that a difference of at least 0.01 CFI units is required to suggest a substantial fit increase [62], results suggest that the magnitude of fit increase for the alternative model was nominal. Given the negligible difference and the stronger theoretical grounding of the original model, we retained the original model as the more parsimonious model.

## 4. Discussion

### 4.1. Overall Discussion

The present study examined whether IU mediates the relationship between social anxiety and PSU severity. Unlike prior research that has largely examined simple associations or relied on cross-sectional correlations, we used SEM to test the mediational pathway using latent constructs, providing a more rigorous analysis of underlying cognitive–affective mechanisms. Our findings supported the main hypotheses, indicating that people with higher levels of social anxiety tend to report greater IU, which in turn is associated with greater PSU severity. These results extend previous mediation studies by demonstrating that IU functions as a latent, mechanism-level mediator linking social anxiety to PSU, offering stronger evidence for the role of IU as a psychological vulnerability contributing to maladaptive technology use. Our study provides a clearer understanding of how individual cognitive–affective processes contribute to PSU.

Our findings are consistent with previous studies indicating that socially anxious people tend to experience higher levels of IU, which can drive maladaptive coping behaviors including excessive smartphone use, as an attempt for reducing these negative emotions and anxiety [25,34,36,63]. This view is consistent with Brown and Medcalf-Bell’s work, which reported that individuals with elevated social anxiety had greater IU, and stronger motives to use phones to decrease their anxiety, which in turn increased smartphone use [19].

From a theoretical standpoint, the findings reinforce the transdiagnostic role of IU in problematic behaviors [64]. The I-PACE model [41,42] suggests that predispositional factors such as social anxiety influence problematic Internet use through cognitive and affective mechanisms. Our study highlights IU as one such mechanism, providing empirical support for the model’s emphasis on individual differences and their impact on technology-related excessive behaviors. Socially anxious individuals often perceive social interactions as threatening and unpredictable, prompting them to rely on smartphone-based communication as a strategy to manage uncertainty [19]. While this behavior may provide short-term relief from uncertainty, it likely reinforces avoidance of face-to-face interaction and leads to dependence on the smartphone, thereby contributing to PSU [65,66].

Although we also examined an alternative model that included a direct path from social anxiety to PSU, the improvement in model fit was minimal. The difference in CFI between the two models was only 0.005, well below the 0.01 threshold typically used to indicate meaningful improvement [40,62]. Therefore, while the alternative model was statistically superior in a narrow sense, the theoretical value and parsimony of the original model remain stronger. As mentioned before, according to the I-PACE framework [41,42] predispositional factors like social anxiety may lead to PSU through cognitive–affective mechanisms, rather than being represented by only a single direct path. Keeping the mediation model aligns with prior research that identifies IU as a key process linking social anxiety to maladaptive smartphone use [19,34,64]. Therefore, keeping the original model of our study was more consistent with previous research and theory.

Our findings also carry important practical implications. Clinically, interventions that focus on reducing IU could help reduce PSU among socially anxious individuals. For instance, cognitive–behavioral strategies targeting IU could be used for socially anxious young adults, a group particularly vulnerable to PSU [64,67,68]. In educational settings, prevention programs could incorporate modules that teach students strategies for managing uncertainty in both offline and online contexts, fostering digital resilience and healthier technology use [69,70]. From a policy perspective, our findings support the development of guidelines that emphasize the early detection of at-risk individuals and promote evidence-based interventions targeting psychological vulnerabilities such as IU [64].

Our study emphasizes the importance of integrating psychological and behavioral mechanisms into personalized approaches to mental healthcare and digital well-being. Our findings contribute to the growing field of personalized and precision mental health by emphasizing the role of individual psychological mechanisms in maladaptive Internet use. Identifying IU as a key mediator between social anxiety and PSU severity highlights how specific cognitive–affective vulnerabilities may increase susceptibility to excessive or maladaptive engagement with digital technologies [41]. Such insights align with the principles of precision mental health, which aim to tailor prevention and intervention strategies based on individual cognitive and emotional profiles [71].

### 4.2. Limitations

Several limitations should be considered. First, because we used a cross-sectional design, causal conclusions cannot be drawn, and future longitudinal or experimental research is needed to confirm IU’s role as a mediator over time [72]. Second, reliance on self-report measures can lead to potential biases, including social desirability and shared method variance; therefore, objective assessments of smartphone use would provide more accurate and reliable data [73]. Third, this study focused specifically on IU as a mediator, whereas the I-PACE model emphasizes multiple interacting mechanisms [41]. Also, we did not analyze depression or general anxiety measures. Future studies should examine how IU interacts with other factors, such as emotion regulation difficulties, fear of missing out, or executive functions, to provide a more comprehensive account of PSU or other forms of Internet misuse [74,75,76]. The current sample consisted primarily of university students from a Midwestern USA State, thereby limiting the generalizability of the findings to wider populations. Future research should investigate these pathways across culturally diverse samples, as cultural norms surrounding uncertainty and technology use may shape both the expression of social anxiety and reliance on smartphones [77]. In particular, collectivist versus individualist orientations may influence the relationship between IU, social anxiety, and PSU, and future studies should examine these potential cultural moderators [78].

### 4.3. Conclusions

In conclusion, the current study demonstrates that IU serves as a mediator in the relationship between social anxiety and PSU, consistent with the I-PACE framework [41,42]. By identifying IU as a key mechanism, this research advances theoretical knowledge of PSU and highlights practical directions for interventions aimed at reducing both social anxiety and PSU.

## Figures and Tables

**Table 1:** Correlations for age and scale scores.

Variable	1	2	3
1. Age			
2. PSU	−0.12 *		
3. IU	0.03	0.43 **	
4. Social Anxiety	−0.08	0.44 **	0.58 **

Note. IU = intolerance of uncertainty, PSU = problematic smartphone use. * indicates *p* < 0.05. ** indicates *p* < 0.01.

**Table 2:** Analyses of variance result for Sex differences in scale scores.

Variable	Women M	Women SD	Men M	Men SD	F(1325)	*p*	η^2^p
IU	31.57	10.26	28.44	9.15	7.44	<0.01	0.02
PSU	28.05	10.29	23.50	8.06	16.79	<0.001	0.05
Social Anxiety	22.86	15.22	15.94	10.76	18.59	<0.001	0.05

Note. M and SD denote the mean and standard deviation, respectively. IU = intolerance of uncertainty, PSU = problematic smartphone use.

**Table 3:** Results of individual confirmatory factor analyses.

Measure	Chi-Square Test	CFI	TLI	RMSEA (90% CI)	SRMR
PSU	χ^2^(33) = 222.84, *p* < 0.001	0.94	0.92	0.13 (0.12–0.15)	0.05
Social Anxiety	χ^2^(115) = 422.81, *p* < 0.001	0.96	0.95	0.09 (0.08–0.10)	0.05
IU	χ^2^(54) = 260.05, *p* < 0.001	0.95	0.94	0.11 (0.10–0.12)	0.04

Note. PSU = problematic smartphone use; IU = intolerance of uncertainty; TLI = Tucker–Lewis Index; CFI = comparative fit index; CI = confidence interval; RMSEA = root mean square error of approximation; SRMR = standardized root mean square residual. All analyses in this table were conducted using robust weighted least squares estimation with a mean- and variance-adjusted chi-square.

**Table 4:** Standardized estimate for the indirect effect of IU in the relationship between social anxiety and PSU.

	Estimate	S.E.	Est./S.E.	Two-Tailed *p*
Indirect Effect of IU (mediator) from social anxiety (predictor) to PSU (dependent variable)	0.38	0.05	8.10	<0.001

Note. IU = intolerance of uncertainty; PSU = problematic smartphone use; S.E. = standard Error.

## Data Availability

The de-identified data from this study are available for download and use, using the following link: https://data.mendeley.com/datasets/sp3h5k27xk/1.

## Appendix A

**Table A1:** Single-factor problematic smartphone use model: standardized estimates for factor loadings (top) and residual error covariances (bottom).

SAS-SV Item	Estimate	S.E.	Est./S.E.	Two-Tailed *p*-Value
1	0.56	0.04	13.10	<0.001
2	0.65	0.04	18.31	<0.001
3	0.59	0.04	14.72	<0.001
4	0.66	0.03	20.83	<0.001
5	0.84	0.02	37.51	<0.001
6	0.86	0.02	46.02	<0.001
7	0.74	0.03	26.40	<0.001
8	0.74	0.03	25.20	<0.001
9	0.64	0.03	19.25	<0.001
10	0.64	0.04	17.57	<0.001
	**Estimate**	**S.E.**	**Est./S.E.**	**Two-Tailed** ***p*** **-Value**
Item 1 with 2	0.27	0.06	4.71	<0.001
Item 4 with 5	0.21	0.06	3.69	<0.001

Note: SAS-SV = Smartphone Addiction Scale-Short Version; S.E. = Standard Error; Est./S.E. = Estimate divided by Standard Error.

## Appendix B

**Table A2:** Single-factor social anxiety model standardized estimates for factor loadings (top) and residual error covariances (bottom).

SPIN Item	Estimate	S.E.	Est./S.E.	Two-Tailed *p*-Value
1	0.58	0.04	13.57	<0.001
2	0.63	0.04	16.51	<0.001
3	0.79	0.03	30.51	<0.001
4	0.74	0.03	25.55	<0.001
5	0.74	0.03	26.82	<0.001
6	0.84	0.02	40.48	<0.001
7	0.72	0.03	23.48	<0.001
8	0.64	0.04	17.57	<0.001
9	0.76	0.03	28.79	<0.001
10	0.79	0.02	33.49	<0.001
11	0.69	0.03	20.99	<0.001
12	0.73	0.03	23.74	<0.001
13	0.73	0.04	21.11	<0.001
14	0.87	0.02	52.19	<0.001
15	0.82	0.02	42.36	<0.001
16	0.72	0.04	20.03	<0.001
17	0.77	0.03	26.11	<0.001
	**Estimate**	**S.E.**	**Est./S.E.**	**Two-Tailed** ***p*** **-Value**
Item 1 with 16	0.49	0.05	9.31	<0.001
Item 5 with 12	0.41	0.05	9.15	<0.001
Item 3 with 8	0.45	0.05	8.37	<0.001
Item 4 with 10	0.35	0.05	6.87	<0.001

Note: SPIN = Social Phobia Inventory; S.E. = Standard Error; Est./S.E. = Estimate divided by Standard Error.

## Appendix C

**Table A3:** Single-factor intolerance of uncertainty model standardized estimates for factor loadings.

IUS Item	Estimate	S.E.	Est./S.E.	Two-Tailed *p*-Value
1	0.72	0.03	24.43	<0.001
2	0.66	0.03	19.93	<0.001
3	0.78	0.03	31.72	<0.001
4	0.69	0.03	24.01	<0.001
5	0.78	0.02	33.01	<0.001
6	0.82	0.02	35.20	<0.001
7	0.82	0.02	39.13	<0.001
8	0.64	0.04	18.24	<0.001
9	0.73	0.03	23.45	<0.001
10	0.72	0.03	24.03	<0.001
11	0.63	0.03	18.74	<0.001
12	0.75	0.03	28.29	<0.001

Note: IUS = Intolerance of Uncertainty Scale-Short version; S.E. = Standard Error; Est./S.E. = Estimate divided by Standard Error.
